# Do Differences in Work Disability Duration Between Men and Women Vary by Province in Canada?

**DOI:** 10.1007/s10926-018-9819-1

**Published:** 2018-11-30

**Authors:** Robert A. Macpherson, Mieke Koehoorn, Jonathan Fan, William Quirke, Benjamin C. Amick, Allen Kraut, Cameron A. Mustard, Christopher B. McLeod

**Affiliations:** 1 Partnership for Work, Health and Safety, School of Population and Public Health, University of British Columbia, Vancouver, BC Canada; 2 Institute for Work & Health, Toronto, ON Canada; 3 Department of Health Policy and Management, Robert Stempel College of Public Health and Social Work, Florida International University, Miami, FL USA; 4 University of Manitoba, Winnipeg, MB Canada

**Keywords:** Workers’ compensation, Return-to-work, Gender, Rehabilitation, Occupational health

## Abstract

*Purpose* Research has shown that there are important sex and gender-based differences in the work disability duration of men and women. This research is often limited to single jurisdictions, using different outcome measures, and therefore has limited generalisability of findings. This study examined if differences between work disability of men and women differed by province and by duration of work disability. *Methods* Cohorts of injured workers in the Canadian provinces of British Columbia (BC), Manitoba (MB) and Ontario (ON) were analysed using workers’ compensation data for work-related injuries occurring between 2007 and 2011. Work disability duration was measured using cumulative days in receipt of disability benefits paid during one-year post-injury. Poisson models with restricted cubic splines tested whether differences between men and women in the likelihood of transitioning off disability benefits varied by duration of work disability in each province, adjusting for confounders. *Results* Men transitioned off disability benefits faster than women for claim durations of up to two to four months, after which women transitioned off disability benefits faster until ten months. Differences between men and women were consistent across all jurisdictions. For claims longer than ten months, men transitioned off work disability benefits faster than women in BC and ON, but there were no significant differences between men and women in MB. *Conclusions* Differences in the work disability duration between men and women vary by province and duration of work disability. Claims management processes need to be sensitive to differences that men and women face and the timing of interventions.

**Electronic supplementary material:**

The online version of this article (10.1007/s10926-018-9819-1) contains supplementary material, which is available to authorized users.

## Introduction

A growing body of literature has identified that there are important differences in the work disability duration of men and women, evident in different likelihoods of injured workers returning to work [1], transitioning off disability benefits [2, 3], and transitioning onto permanent disability pension [4–6]. Understanding why differences may exist between men and women’s occupational health outcomes, such as work disability duration, is challenging due to the role of biological sex-based factors as well as more socially determined gender-based factors. Applying a “gender perspective” has been encouraged to try and grasp the complex relationships between sex, work-related factors and social circumstances (family relations, employment status, and social class) [7]. Stratification of analyses by men and women has been proposed as a method to account for differences in occupational health outcomes by sex and gender [8]. Nonetheless, it has been argued that such an approach more appropriately captures sex-based differences rather than gender [9, 10], prompting researchers to create indexes to measure gender based on other observable characteristics when no direct measure of gender is available [11].

A limitation of existing sex and gender based research on work disability has been the tendency to focus on single workers’ compensation jurisdictions, overlooking the possibility that differences between men and women may vary by jurisdiction. This is despite the fact that, compared to sex, gender is socially constructed and therefore its effects are potentially varying by jurisdictional context. Applying a comparative approach to analysing differences in disability durations and return-to-work (RTW) between men and women offers an approach to tease apart what differences may be sex-based and what may be gender-based.

The extent of observed gender differences in work disability duration can vary by context and methodology. Research using survey and interview data in the Netherlands found that, among workers with at least one month off work due to mental and musculoskeletal conditions, women were significantly less likely to experience lasting RTW (RTW without relapse) [1]. In Norway, administrative data were used to examine the likelihood of workers with long-term sickness absence (≥ 8 weeks) transitioning onto permanent disability to find that despite larger proportions of permanent disability among women (6.5%) than men (4.9%), there was no statistically significant difference after adjusting for confounders [4]. In Quebec, Canada, using administrative data, men and women transitioned off work disability benefits at a similar rate in the first 3-to-12 months but by the second and third years, the transition slowed more for men, resulting in a statistically significant shorter duration for women over the long term [2].

Individuals’ labour market and health trajectories are embedded within work and social contexts that shape their trajectories [12]. In terms of RTW following work-related injury, the experience of an injured man or woman may be shaped not only by their individual, family or work conditions, but also by the jurisdictional workers’ compensation and health care systems. For example, research has shown that the role of doctors presented an important jurisdictional difference between Ontario and Quebec, with doctors from the latter province having the final decision in the assignment of modified work during recovery process for injured workers [13]. Health services research has shown that compared to men, women are more likely to visit health care providers [14], and are more likely to receive prescriptions for all types of drugs, including opioids [15].

Multi-jurisdictional studies of work disability duration tend to focus on gender as a determinant of work disability without explicitly examining how the gender-RTW relationship may be modified across jurisdictional contexts. For example, research in Australia found that women were significantly less likely to transition off work disability benefits during a two-year follow-up, post-injury after accounting for jurisdictional-level variables [3]. A study of low back pain across multiple jurisdictions in the United States found that the mean length of work disability was 1.9 days greater among women compared to men, after adjusting for state-level variables such as wage replacement rate, waiting periods, treatment providers, and medical fees [16]. Neither of these studies examined how the differences between men and women vary by jurisdiction.

Much of prior sex and gender-based research conducts separate analyses for men and women, enabling researchers to identify whether certain predictors differ by gender or sex [17]. A limitation of this is that few studies can state whether the effect of sex or gender itself is significant and whether it changes depending on the duration of work disability. No studies have specifically set out to examine whether gender differences in work disability vary by the duration of work disability. However, as illustrated in the Quebec study, there were differences in the rate at which men and women transitioned off benefits according to the phase of recovery (i.e., shorter versus longer time since injury) [2]. This suggests that differences between men and women may vary depending on phase of disability (e.g., acute vs. subacute/chronic), in the same way that injury severity or previous claim history has been shown to in previous studies [18, 19]. Since the Quebec study did not model the differences between men and women while adjusting for potential confounders, it remains to be seen whether such differences persist after adjusting for factors such as age, occupation, and injury type. The authors of the Quebec study also stated in their conclusions that future studies should investigate whether their findings are generalizable to other jurisdictions. Based on these gaps in the literature, the two research questions of this paper are: (1) Do differences between the likelihood of men and women transitioning off disability benefits vary by province? (2) Do differences between the likelihood of men and women transitioning off disability benefits vary by duration of work disability?

## Methods

### Data

Claim-level data from three provincial workers’ compensation systems in Canada were used for this study: WorkSafeBC (Workers’ Compensation Board of British Columbia), WCB Manitoba (Workers Compensation Board of Manitoba), and WSIB Ontario (Workplace Safety and Insurance Board of Ontario). In Canada, workers’ compensation benefits are administered under statutory, no-fault systems that provide coverage for wage-loss benefits, permanent disability benefits, dependency benefits, and rehabilitation via employer-paid insurance premiums. Each jurisdiction shares core features and mandates, although there are some differences such as coverage (e.g., industries covered), benefits (e.g., maximum insurable earnings), rehabilitation (e.g., early RTW), healthcare (e.g., access to coverage), administration (e.g., appeal procedures) and job protection (e.g., employer obligations to accommodate injured workers) [20].

The average workforce coverage during our study period was 94% for British Columbia (BC), 72% Manitoba (MB), and 72% for Ontario (ON) [21]. Data access, extraction and linkage services were provided by Population Data BC. Use of the data for research purposes was governed by an agreement between the data stewards and the researchers. Personal identifiers were removed from the data provided to the researchers and replaced with an anonymous claim identifier [22, 23]. Ethical approval for the research project was obtained from the Behavioural Research Ethics Board at the University of British Columbia (# H13-00132). Data analysis was conducted using Stata 14.0 (StataCorp, College Station, TX).

### Cohort Criteria

Comparable cohorts were created based on similar claim eligibility and follow-up across the three jurisdictions. Eligible claims included non-fatal injuries that occurred between the years 2007 and 2011, with at least one day of work disability payments post-injury. The time restriction allowed follow-up of claims for at least one year, post-injury. Claims based on occupational diseases (such as asthma and cancer) were excluded due to differences in coverage and processing/timing between exposure onset and claim registration, in comparison to occupational injury claims. Inclusion criteria focused on individuals aged 15–89 years during the time of injury.

Injury data were coded across jurisdictions using the Canadian Standards Association (CSA) Z795-03 standard [24]. Injury types were defined based on research using similar data [25], resulting in two broad injury groupings: strain and non-strain (acute) injuries. Strain injuries included strains and sprains of the back, and other areas (upper and lower limbs, including bursitis, tendinitis, or tenosynovitis). Non-strain injuries included fractures, concussions and other injuries (amputations, dislocations, cold and heat exposures, burns abrasions, contusions, and lacerations). Injury year was also included to capture period effects reflecting differing labour market conditions and jurisdictional workers’ compensation policies. Occupation was defined based on the three-digit National Occupational Classification 2006 code [26].

Work disability duration was measured using cumulative days that claims received disability benefit payments, right-censored at 260 days (equivalent to one year based on a five-day work week). This measure differs to calendar days elapsed between date of injury and last day of receiving disability benefits as it included consecutive and non-consecutive disability days. This measure has been used in similar studies using administrative data when calendar RTW event data are unavailable [2, 3]. Benefit payments for vocational rehabilitation, health care, and long-term disability were excluded due to these likely being associated with different work disability and recovery processes compared to short-term disability in which a worker can return to their previous job.

### Statistical Analyses

Regression analyses were initially conducted using Cox proportional hazards models. However, the underlying proportionality assumptions were violated. While other research using similar data have dealt with non-proportional hazards by using piecewise models [27], a limitation of this approach is the arbitrary choice of where to partition the time axis. Poisson models with restricted cubic splines (RCS) were used to overcome this issue by creating smoothed functions of time that could be interacted with gender to estimate the time-varying effects of gender while adjusting for confounders including age, injury type, injury year, and occupation [28].

Using the estimates of the RCS coefficients and interactions, it was possible to obtain an estimate of the log baseline hazard function to calculate and graph the hazard ratio (HR) with 95% confidence intervals (CI) of women transitioning off disability benefits, compared to men [28]. To examine variations in HRs over time and across jurisdictions, we estimated models for each jurisdiction using varying numbers and locations of knots for the restricted cubic splines. Akaike information criterion was used to identify the best fitting models, which were based on five degrees of freedom (two boundary knots and three interior knots based on the 25th, 50th, 75th and 90th centiles of uncensored survival times). Models were created for all injuries and occupations in each of the jurisdictions, followed by separate models for strain and back strain injuries due to hypothesized greater gender differences based on the literature [29, 30].

## Results

Table 1 shows the distribution of key study variables, stratified by jurisdiction and gender. The BC cohort included 258,246 claims, and the MB and ON cohorts included 69,941 and 287,556 claims, respectively. The majority of claims in each jurisdiction were from men, contributing 66% claims in BC, 67% in MB, and 61% in ON. The average work disability days paid was higher for men in BC and ON, and higher for women in MB. The average age of workers at the time of injury was similar across jurisdiction, with an older age among women than men. Strains (the most common injuries) were higher among women than men whereas non-strain injuries (excluding concussion) were higher among men. The frequency of injury claims decreased over time for men and women in all jurisdictions. Across all three jurisdictions, injuries among men were more common in trades, transport and equipment operators and related occupations, whereas injuries among women were more common in sales and service occupations.

**Table 1:** Descriptive statistics of study cohort by province and gender

	BC		MB		ON	
	Men	Women	Men	Women	Men	Women
	(n = 170,330)	(n = 87,916)	(n = 46,972)	(n = 22,969)	(n = 174,353)	(n = 113,203)
Work disability days (mean)	34.84	33.60	27.31	31.86	33.19	27.03
Age (mean)	39.45	41.96	38.85	42.17	40.77	42.78
Injury type						
Strain injuries	57.12	68.00	61.76	73.64	57.53	64.51
Back strains	23.38	24.42	26.30	28.70	25.54	25.84
Other strains	33.74	43.58	35.46	44.94	31.99	38.67
Non-strain injuries	42.89	32.00	38.24	26.35	42.47	35.49
Fractures	8.39	4.91	6.60	4.05	9.79	6.99
Concussions	2.23	2.75	0.63	0.68	1.14	1.53
Other injuries	32.27	24.34	31.01	21.62	31.54	26.97
Injury year						
2007	24.06	21.48	22.33	20.98	24.29	22.96
2008	23.38	22.33	22.19	21.32	23.08	22.80
2009	17.82	18.57	19.95	20.45	18.72	19.60
2010	17.14	18.46	17.55	19.36	17.30	17.80
2011	17.61	19.16	17.98	17.89	16.61	16.84
Occupation						
Management	1.54	2.69	1.20	2.33	1.57	3.39
Business, finance, administration	3.71	6.39	4.40	7.38	6.35	11.01
Natural and applied sciences and related	1.88	0.64	1.54	0.98	2.09	0.95
Health	2.11	23.55	2.59	32.06	2.50	20.16
Social science, education, govt. service, religion	0.98	7.41	0.86	6.83	1.51	9.56
Art, culture, recreation, sport	0.99	1.88	0.32	0.72	0.49	0.90
Sales, service	14.99	42.64	14.80	35.21	21.38	38.08
Trades, transport and equipment operators and related	58.28	7.40	57.73	6.83	45.73	6.19
Unique to primary industry	5.14	2.12	2.48	0.81	2.85	0.97
Unique to processing, manufacturing, utilities	10.38	5.28	14.07	6.85	15.53	8.81

Numbers in table are proportions unless otherwise distinguished

*BC* British Columbia, *MB* Manitoba, *ON* Ontario

The coefficients of the Poisson models with RCS are shown in Online Resource 1. Although there is no direct interpretation of the RCS and interaction coefficients [28], the sign and significance of the estimates suggest that there was both a difference in the ratio of hazards between men and women and that this varied over time. Table 2 summarizes the HRs at specific points in time, estimated from the Poisson models. In addition to the differences between men and women transitioning off disability benefits persisting even after adjusting for a range of individual characteristics, these differences varied depending on the duration of disability. Overall, women had lower likelihoods of transitioning off disability benefits than men for durations of up to approximately 2-to-4 months, after which they had higher likelihoods until around 10 months. This was relatively consistent by jurisdiction and injury type.

**Table 2:** Time-dependent hazard ratios for women transitioning off work disability benefits compared to men, by injury type and province

	All injuries					
	BC		MB		ON	
	HR	95% CI	HR	95% CI	HR	95% CI
1 day	0.94	0.91–0.96	0.96	0.92–1.00	1.07	1.05–1.09
1 week	0.83	0.81–0.84	0.88	0.85–0.91	0.93	0.91–0.94
1 month	0.92	0.90–0.93	0.85	0.81–0.88	0.87	0.85–0.89
2 months	1.05	1.03–1.07	0.91	0.87–0.95	0.92	0.90–0.94
3 months	1.13	1.10–1.15	0.99	0.90–1.03	1.01	0.99–1.03
6 months	1.09	1.05–1.13	1.12	1.05–1.20	1.17	1.13–1.21
9 months	0.99	0.96–1.02	1.13	1.06–1.20	1.05	1.03–1.08
12 months	0.88	0.83–0.94	1.07	0.93–1.22	0.83	0.79–0.88

	Strain injuries					
	BC		MB		ON	
	HR	95% CI	HR	95% CI	HR	95% CI
1 day	0.97	0.94–1.00	1.02	0.96–1.07	1.09	1.06–1.11
1 week	0.76	0.74–0.77	0.85	0.81–0.88	0.85	0.84–0.87
1 month	0.87	0.85–0.89	0.80	0.76–0.84	0.84	0.82–0.86
2 months	1.07	1.05–1.10	0.92	0.87–0.97	0.93	0.90–0.95
3 months	1.17	1.14–1.20	1.02	0.96–1.07	1.01	0.98–1.04
6 months	1.08	1.04–1.13	1.11	1.03–1.20	1.08	1.04–1.13
9 months	0.99	0.95–1.03	1.11	1.03–1.20	1.00	0.97–1.04
12 months	0.91	0.83–0.98	1.07	0.91–1.25	0.86	0.80–0.92

	Back strain injuries					
	BC		MB		ON	
	HR	95% CI	HR	95% CI	HR	95% CI
1 day	0.95	0.90–0.99	1.02	0.94–1.12	1.06	1.02–1.10
1 week	0.73	0.70–0.76	0.80	0.75–0.86	0.79	0.77–0.81
1 month	0.80	0.77–0.83	0.72	0.66–0.77	0.82	0.79–0.85
2 months	1.02	0.98–1.07	0.89	0.81–0.96	0.97	0.92–1.01
3 months	1.17	1.12–1.22	1.00	0.92–1.08	1.09	1.04–1.14
6 months	1.20	1.12–1.29	1.09	0.96–1.23	1.17	1.10–1.25
9 months	1.05	0.97–1.14	1.05	0.92–1.19	1.03	0.97–1.09
12 months	0.85	0.71–1.02	0.95	0.72–1.25	0.82	0.73–0.91

Time-dependent hazard ratios were estimated from a Poisson model incorporating restricted cubic splines where the time-dependent effect is a linear function of time. Models were adjusted for age, injury type, injury year, and occupation

*BC* British Columbia, *MB* Manitoba, *ON* Ontario, *HR* hazard ratio, *CI* confidence interval

**Fig. 1:**
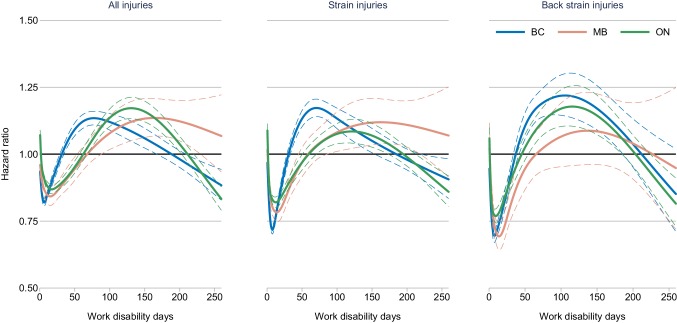
Estimated time-dependent hazard ratios of women transitioning off work disability benefits compared to men, by injury type and province^a^. *BC* British Columbia, *MB* Manitoba, *ON* Ontario. ^a^Dashed lines indicate 95% confidence intervals. Models adjusted for age, injury type, injury year, and occupation

Figure 1 plots the HRs for the complete disability duration of each stratified model. In the model including all injury types, women had a significantly lower likelihood of transitioning off disability benefits compared to men, during the initial phases of the work disability timeline. Over time, the difference between men and women in the likelihood of transitioning off disability benefits changed. In BC, from around 7 days, the difference in the likelihood of transitioning off work disability benefits was lower among women (HR 0.82; 95% CI 0.81, 0.84) until 35 days, after which women became more likely to transition off disability benefits (HR 1.01; 95% CI 1.00, 1.03). The greater likelihood of women transitioning off disability benefits peaked at around 78 days (HR 1.13; 95% CI 1.11, 1.16) before declining until 210 days, when women once again had lower likelihoods than men (HR 0.97; 95% CI 0.93, 1.00). In MB and ON, a similar time-varying effect of gender was observed, with women being significantly more likely to transition off disability benefits at 86 days in MB (HR 1.05; 95% CI 1.00, 1.10) and 68 days in ON (HR 1.02; 95% CI 1.00, 1.05). Women in ON presented the greatest likelihood of transitioning off disability benefits compared to men at 131 days (HR 1.17; 95% CI 1.13, 1.21), before declining on a steeper gradient than BC. In contrast, in MB, the likelihood of women transitioning off disability benefits continued to remain higher than that of men, but presented no significant differences after 233 days (HR 1.10; 95% CI 0.99, 1.21).

For strain injuries, the extent of gender differences in transitioning off disability benefits were greater in BC than the other jurisdictions, with women significantly less likely at day 3 (HR 0.72; 95% CI 0.71, 0.74), and significantly more likely at day 34 (HR 1.03; 95% CI 1.00, 1.05), peaking at day 71 (HR 1.17; 95% CI 1.14, 1.21). In ON, the difference between men and women remained smaller than the model for all injuries. In MB, the results were similar to the all injury model.

For back strain injuries, we observed greater differences between men and women transitioning off disability benefits across jurisdictions, as well as differences across time periods. The greatest differences were observed in BC, with women 30% less likely to transition off disability benefits at 8 days (HR 0.70; 95% CI 0.67, 0.72) and 22% more likely at 106 days (HR 1.22; 95% CI 1.14, 1.30). In ON, women were initially more likely to transition off benefits compared to men (HR 1.06, 95% CI 1.02, 1.10), then less likely at 5 days (HR 0.79, 95% CI 0.77, 0.81), most likely at 116 days (HR 1.18, 95% CI 1.10, 1.22) and finally, less likely at 227 days (HR 0.92, 95% CI 0.86, 0.99). In MB, significant gender differences were only observed for claims up to 44 days (HR 0.91, 95% CI 0.84, 0.99), with women being less likely to transition off benefits.

## Discussion

This study examined whether differences in work disability between men and women were consistent by provincial compensation jurisdiction and duration of disability. There are two main findings. Firstly, after adjusting for confounders such as age, occupation, injury type and injury year, differences in men and women’s work disability duration persisted in all three jurisdictions. Secondly, the differences between men and women varied by duration of disability in all three jurisdictions. In BC, men were more likely to transition off disability benefits than women for claims of less than two months and more than ten months. In ON, with the exception of claims of only one day, a similar pattern was observed to BC. In MB, men were more likely to transition off disability benefits until around four months and less likely to until around ten months, after which no significant differences between men and women remained.

On the one hand, there was a degree of consistency in the findings across the provinces. This may be reflective of the similarities in the demographics and structure of the labour force, as well as the study cohorts. On the other hand, although the overall patterns of hazard ratios for men and women were similar across province, there were variations in the magnitude and timing of differences between men and women in transitioning off disability benefits. This suggests that differences in health care systems and compensation systems across provinces, and gendered factors, may be important factors behind differences between men and women’s disability duration. For instance, it may be that jurisdictional differences in health care services, such as the role of doctors, interacts with gendered differences in the likelihood of visiting health care providers [13, 14]. These differences could potentially impact the likelihood that an injured worker is offered modified duties. While a study in Ontario found no significant gender differences in the likelihood of being offered or accepting work accommodation (commonly referred to as modified work) [31], no study to our knowledge has examined whether this is the case in all Canadian provinces.

The finding of differences varying over time between men and women is consistent with previous research. In Quebec, men and women transitioned off benefits at a similar rate in the first three to twelve months but slowed for men in the two to three year period [2]. Long durations away from work for the Quebec cohort may have had more negative effects on men than women as an explanation for lower likelihoods of men transitioning off benefits between two and ten months. For example, previous studies have found that unemployment had more of an effect on the mental health of men compared to women [32], and that men were less likely to experience RTW with mental complaints and long-term diseases than musculoskeletal complaints [1]. Another possible explanation for the time-varying differences between men and women is the severity of their injuries. Research on workers with low back injury in the U.S. state of California found RTW rates were five times higher for workers with less severe injuries during the acute phase of disability (≤ 30 days) but around two times higher during the subacute/chronic phase of disability (> 30 days) [18]. The authors also found that physical and psychosocial job demands were significant predictors of RTW at all phases of disability but previous lost-time back injuries were associated with greater RTW in the subacute/chronic phase. Although the cohorts of this study were restricted to traumatic work-related injuries, there may be differences between the severity of men and women’s injury and also undiagnosed mental health issues that explain the observed differences over time in the current study.

### Strengths and Limitations

Comparative studies of gender differences in work disability duration are limited in number. Due to studies in different jurisdictions applying different cohort criteria, and different measures, it is difficult to draw conclusions as to whether differences observed in one jurisdiction will be generalizable to other jurisdictions. A unique contribution of this study has been the ability to examine gender differences across multiple jurisdictions using consistent methodology and similar data sources. In doing so, the study has shown that in addition to there being a consistent time-varying effect in the difference between men and women across jurisdiction, the size of the effect was modified by jurisdiction. The fact that there were differences in the effect size across the jurisdictions suggests that rather than simply being sex-based differences, this study captures gender-based differences as these are socially constructed differences that may be contextualized differently in each jurisdiction [2].

Had the study cohorts been pooled without examining gender differences in each jurisdiction, the main finding of gender differences by jurisdiction and by duration of disability would not have been identified. Furthermore, had standard Cox proportional hazards models been used, the results of this study would have shown women to be significantly less likely to transition off disability benefits than men along all points of the distribution of disability days. While such a finding would be consistent with the literature [1, 3], these averaged effects would bias the results, especially for claim durations of two to ten months. Additionally, by using Poisson models with RCS, this study provides improved time-varying HR estimates from previous studies that have relied on more arbitrary cut-offs in time [27].

Although the cohorts were restricted to claims receiving short-term disability benefit payments during the first year post-injury, a limitation of the outcome variable (cumulative disability days until transitioning off benefits) is that it could have potentially captured transitions onto vocational rehabilitation, permanent disability benefits, non-RTW, or receipt of no benefits. While research on long-term sickness absence and transitions to permanent disability have shown no significant gender differences after adjusting for confounders [6], and research on the offer and acceptance of modified RTW has also shown no gender differences [31], no studies have examined how these differing outcomes may compete against each other. Another limitation to the outcome variable is that, because it is a cumulative measure of disability days, while one could be comparing two injured workers with the same disability days paid, one of them may have accumulated the days over a longer calendar period than the other. Both these limitations could be addressed in future research by collecting and examining detailed RTW event data across multiple jurisdictions. This would enable the calculation of calendar days between the date of injury and the date of different outcomes, such as modified RTW, full RTW, vocational rehabilitation, and permanent disability. Doing so would provide a greater understanding to the findings of this study and gender and jurisdictional differences in the RTW process more generally.

A contributing factor to gender differences in work disability duration is gender segregation in the labour market, both horizontally (across industry sectors) and vertically (within the occupational hierarchy). The models in this study adjusted for broad occupation groups. A limitation of this is that it only captured some of the gender segregation in terms of industry sector, compared to what could have been captured had a common industry classification been available at the time of the study. Furthermore, despite using a three-digit classification, this may not have fully accounted for differences in the work demands or duties of men and women within the same professions. For example, previous research has found that, even when comparing men and women with the same job titles, they may carry out different tasks and therefore be at differing risks of injury [33]. By the same logic, men and women with the same jobs may experience differing rates of injury recurrence and RTW. Future comparative gender research would undoubtedly benefit from more detailed data regarding job characteristics, not only including the job title but also job duties.

## Implications

One of the main implications of this study is that it reinforces the importance of considering gender differences in work disability duration. Reflecting the conclusions of similar work, efforts should be made by workers’ compensation boards and employers to tailor disability prevention and management efforts to men and women’s specific needs and barriers [2]. Given the significant gender-by-time interaction on work disability duration identified in this study, the timing of disability management interventions should also be given consideration. In shorter claim durations (less than two to four months), women may require additional attention to RTW, whereas for longer claim durations (four to ten months), men may require additional attention.

Another important policy implication of our findings is that while there was an overall consistency across jurisdictions, the gender differences also changed depending on the duration of work disability. For example, women in ON were transitioning off disability benefits faster than men for claims of only one day whereas this was not the case in BC and MB. The point at which women were most likely to transition off disability benefits than men peaked earlier in BC before the other two jurisdictions. These findings suggest that system-level jurisdictional characteristics, such as health care and workers’ compensation may play a role in explaining gender differences in work disability duration. While the examination of specific system-level jurisdictional differences on RTW of men and women goes beyond the scope of this initial study, future research should investigate whether disability prevention and management programs in specific jurisdictions can help reduce gender differences in work disability duration.

## Electronic supplementary material

Below is the link to the electronic supplementary material.

Supplementary material 1 (DOCX 17 KB)

## Data Availability

The datasets generated and analysed during the current study are not publicly available. The datasets were accessed under various information sharing agreements adhering to Canadian privacy legislation that impose legal restrictions in the access and use of workers’ compensation data. Furthermore, British Columbia privacy legislation restricts research data to be accessible in Canada only. As this is a comparable study, all the data falls under this legislation.
