# Supplementary material for: Do Differences in Work Disability Duration Between Men and Women Vary by Province in Canada?
Source: J Occup Rehabil. 2018 Nov 30;29(3):560–8. doi: 10.1007/s10926-018-9819-1 (PMC6675772; doi:10.1007/s10926-018-9819-1)
Supplement: Supplementary file 1 — Supplementary material 1 (DOCX 17 KB) [file 10926_2018_9819_MOESM1_ESM.docx]

**Online Resource 1** Time-varying effects of women transitioning off work disability benefits compared to men, by injury type and province^a^

|  | **All injuries** | | | | | | | |
| --- | --- | --- | --- | --- | --- | --- | --- | --- |
|  | BC | |  | MB | |  | ON | |
|  | Coef. | 95% CI |  | Coef. | 95% CI |  | Coef. | 95% CI |
| Women (Ref = men) | 0.01 | (-0.01, 0.02) |  | -0.03 | (-0.06, -0.00) |  | 0.00 | (-0.02, 0.01) |
| rcs1 | -0.31 | (-0.31, -0.30) |  | -0.50 | (-0.52, -0.49) |  | -0.41 | (-0.42, -0.41) |
| rcs2 | -0.43 | (-0.43, -0.42) |  | -0.48 | (-0.49, -0.46) |  | -0.63 | (-0.64, -0.62) |
| rcs3 | 0.18 | (0.18, 0.19) |  | 0.16 | (0.15, 0.18) |  | 0.03 | (0.02, 0.03) |
| rcs4 | 0.20 | (0.20, 0.21) |  | 0.13 | (0.12, 0.14) |  | 0.33 | (0.32, 0.34) |
| rcs5 | -0.03 | (-0.03, -0.03) |  | -0.03 | (-0.04, -0.03) |  | -0.02 | (-0.02, -0.01) |
| Women*rcs1 | 0.04 | (0.02, 0.05) |  | 0.09 | (0.07, 0.12) |  | 0.05 | (0.04, 0.06) |
| Women*rcs2 | 0.09 | (0.08, 0.10) |  | 0.01 | (-0.01, 0.03) |  | 0.03 | (0.02, 0.04) |
| Women*rcs3 | -0.02 | (-0.03, -0.01) |  | -0.05 | (-0.07, -0.02) |  | 0.06 | (0.05, 0.06) |
| Women*rcs4 | 0.02 | (0.01, 0.03) |  | 0.02 | (0.00, 0.04) |  | -0.07 | (-0.08, -0.05) |
| Women*rcs5 | 0.02 | (0.01, 0.02) |  | 0.00 | (-0.01, 0.01) |  | 0.01 | (0.00, 0.01) |
| Constant | -3.34 | (-3.36, -3.31) |  | -3.33 | (-3.38, -3.28) |  | -3.57 | (-3.59, -3.54) |
| N (observations)^b^ | 5,039,830 | |  | 1,497,942 | |  | 5,400,232 | |
|  | **Strain injuries** | | | | | | | |
|  | BC | |  | MB | |  | ON | |
|  | Coef. | 95% CI |  | Coef. | 95% CI |  | Coef. | 95% CI |
| Women (Ref = men) | 0.00 | (-0.02, 0.02) |  | -0.04 | (-0.08, -0.01) |  | -0.03 | (-0.05, -0.02) |
| rcs1 | -0.32 | (-0.33, -0.31) |  | -0.49 | (-0.51, -0.47) |  | -0.39 | (-0.40, -0.38) |
| rcs2 | -0.41 | (-0.41, -0.40) |  | -0.47 | (-0.49, -0.46) |  | -0.64 | (-0.64, -0.63) |
| rcs3 | 0.19 | (0.18, 0.20) |  | 0.18 | (0.16, 0.20) |  | 0.02 | (0.02, 0.03) |
| rcs4 | -0.11 | (-0.11, -0.10) |  | 0.11 | (0.09, 0.12) |  | 0.29 | (0.28, 0.30) |
| rcs5 | 0.16 | (0.15, 0.16) |  | -0.04 | (-0.05, -0.03) |  | -0.03 | (-0.03, -0.02) |
| Women*rcs1 | 0.05 | (0.04, 0.07) |  | 0.10 | (0.08, 0.13) |  | 0.05 | (0.03, 0.06) |
| Women*rcs2 | 0.12 | (0.11, 0.13) |  | 0.02 | (0.00, 0.05) |  | 0.05 | (0.03, 0.06) |
| Women*rcs3 | -0.01 | (-0.03, 0.00) |  | -0.05 | (-0.08, -0.02) |  | 0.06 | (0.05, 0.07) |
| Women*rcs4 | -0.06 | (-0.07, -0.05) |  | 0.05 | (0.03, 0.07) |  | -0.03 | (-0.05, -0.02) |
| Women*rcs5 | 0.00 | (-0.01, 0.01) |  | 0.01 | (-0.00, 0.02) |  | 0.02 | (0.01, 0.02) |
| Constant | -3.31 | (-3.35, -3.28) |  | -3.34 | (-3.40, -3.28) |  | -3.54 | (-3.57, -3.51) |
| N (observations) ^b^ | 2,842,043 | |  | 992,413 | |  | 3,023,249 | |
|  | **Back strain injuries** | | | | | | | |
|  | BC | |  | MB | |  | ON | |
|  | Coef. | 95% CI |  | Coef. | 95% CI |  | Coef. | 95% CI |
| Women (Ref = men) | -0.02 | (-0.05, 0.00) |  | -0.11 | (-0.16, -0.05) |  | -0.01 | (-0.04, 0.02) |
| rcs1 | -0.37 | (-0.39, -0.36) |  | -0.52 | (-0.55, -0.49) |  | -0.45 | (-0.47, -0.44) |
| rcs2 | -0.33 | (-0.34, -0.32) |  | -0.22 | (-0.24, -0.21) |  | -0.67 | (-0.68, -0.66) |
| rcs3 | 0.15 | (0.13, 0.16) |  | 0.42 | (0.39, 0.45) |  | -0.04 | (-0.04, -0.03) |
| rcs4 | -0.06 | (-0.06, -0.05) |  | 0.08 | (0.06, 0.10) |  | 0.29 | (0.28, 0.31) |
| rcs5 | 0.15 | (0.14, 0.16) |  | -0.03 | (-0.05, -0.00) |  | -0.04 | (-0.04, -0.04) |
| Women*rcs1 | 0.11 | (0.09, 0.14) |  | 0.11 | (0.07, 0.16) |  | 0.07 | (0.04, 0.09) |
| Women*rcs2 | 0.12 | (0.10, 0.13) |  | -0.05 | (-0.07, -0.03) |  | 0.08 | (0.06, 0.10) |
| Women*rcs3 | -0.08 | (-0.10, -0.05) |  | -0.09 | (-0.13, -0.05) |  | 0.08 | (0.07, 0.09) |
| Women*rcs4 | -0.07 | (-0.08, -0.05) |  | 0.03 | (-0.00, 0.06) |  | -0.06 | (-0.08, -0.03) |
| Women*rcs5 | 0.01 | (-0.00, 0.03) |  | -0.03 | (-0.07, 0.01) |  | 0.02 | (0.01, 0.02) |
| Constant | -3.31 | (-3.36, -3.26) |  | -3.35 | (-3.44, -3.25) |  | -3.62 | (-3.66, -3.57) |
| N (observations) ^b^ | 989,083 | |  | 380,204 | |  | 1,232,051 | |

*BC* British Columbia, *MB* Manitoba, *ON* Ontario, *HR* hazard ratio, *CI* confidence interval, *RCS* restricted cubic spline. ^a^The coefficients are estimated from a Poisson model incorporating RCSs with 5 degrees of freedom to create a smoothed function of time. ^b^Observations do not match those of Table 1 as the data have been collapsed over each covariate pattern to reduce computational time. Models were adjusted for age, injury type, injury year and occupation.
